# Human ApoE2 Endows Stronger Contractility in Rat Cardiomyocytes Enhancing Heart Function

**DOI:** 10.3390/cells12030347

**Published:** 2023-01-17

**Authors:** Yang Wu, Fujie Zhao, Venkata N. Sure, Abdulgafar Ibrahim, Changjiang Yu, Sean M. Carr, Ping Song

**Affiliations:** Center for Molecular and Translational Medicine, Georgia State University, Atlanta, GA 30303, USA

**Keywords:** human ApoE isoform, rat model, contractile function of the heart, fatty acid β-oxidation, intracellular Ca^2+^ transient

## Abstract

(1) Background: Apolipoprotein E (ApoE) is a critical plasma apolipoprotein for lipid transport and nonlipid-related functions. Humans possess three isoforms of ApoE (2, 3, and 4). ApoE2, which exhibits beneficial effects on cardiac health, has not been adequately studied. (2) Methods: We investigated the cardiac phenotypes of the humanized ApoE knock-in (hApoE KI) rats and compared to wild-type (WT) and ApoE knock-out (ApoE KO) rats using echocardiography, ultrasound, blood pressure measurements, histology strategies, cell culture, Seahorse XF, cardiomyocyte contractility and intracellular Ca^2+^ tests, and Western blotting; (3) Results: hApoE2 rats exhibited enhanced heart contractile function without signs of detrimental remodeling. Isolated adult hApoE2 cardiomyocytes had faster and stronger sarcomere contractility because of more mitochondrial energy generation and stimulation-induced fast and elevated intracellular Ca^2+^ transient. The abundant energy is a result of elevated mitochondrial function via fatty acid β-oxidation. The fast and elevated Ca^2+^ transient is associated with decreased sarcoplasmic reticulum (SR) Ca^2+^ ATPase (SERCA2) and increased expression of cardiac ryanodine receptor 2 (RyR2) conducting a potent Ca^2+^ release from SR.; (4) Conclusions: Our studies validated the association of polymorphic ApoEs with cardiac health in the rat model, and revealed the possible mechanisms of the protective effect of ApoE2 against heart diseases.

## 1. Introduction

Apolipoprotein E (ApoE) is a crucial plasma apolipoprotein mainly involved in lipid and lipoprotein homeostasis. It is derived primarily from the liver and circulating in the plasma, but is also expressed in non-hepatic tissues, including the spleen, adrenal glands, kidney, testes, ovaries, heart, and lungs [1]. In addition to fat metabolism, ApoE is also involved in nonlipid-related functions, such as glucose tolerance and insulin sensitivity, immunoregulation, oxidation, cell proliferation and migration, and extracellular matrix deposition [2,3,4]. Unlike rodent ApoE (only one isoform), human ApoE has three isoforms (E2, E3, and E4), which have differences in amino acids at positions 112 and 158 [5]. ApoE3 is the predominate isoform, with an allelic frequency of 78%, and is considered the normal isoform [6,7]. ApoE2 differs from ApoE3 by the substitution of Arg with Cys at 158 amino acid site, which disrupts the low density lipoprotein receptor (LDLR) recognition site, lowering its binding affinity to LDLR and impairing the clearance of triglyceride-rich lipoprotein remnant particles [8]. ApoE4 has Arg at 112 site instead of Cys (ApoE3), which leads to impaired lipolytic processing, although ApoE4 has similar LDLR binding affinity as ApoE3 [8,9]. Both ApoE2 and ApoE4 are closely associated with disease outcomes. ApoE4 (allelic frequency of 15%) is the major isoform that confers increased susceptibility to elevated VLDL/HDL-cholesterol ratio [10,11], coronary heart disease (CHD) [12], and Alzheimer’s disease (AD) [13,14]. ApoE2 (allelic frequency of 7%) is associated with type III hyperlipoproteinemia (higher cholesterol and triglyceride levels) [15], enhanced longevity [16,17], and decreased susceptibility to AD [18] and CHD [19]. APOE polymorphic alleles are critical genetic determinants of cardiovascular diseases (CVDs), especially CHD [20]. ApoE2 has potential protection against CHD, despite the fact that the ApoE2 carriers are not immune to atherosclerosis [21]. Numerous animal studies attempt to elucidate the effect of isoform ApoE4 on AD risk. However, little attention is paid to the association of human ApoE isoforms and cardiac health using animal models. It is significant to reveal the association of ApoE isoform with cardiovascular health and the potential mechanisms of the protective role of ApoE2 against CHDs using animal models.

Humanized ApoE knock-in (hApoE KI) rats recently became available, in which the endogenous rat ApoE gene was replaced by different human *APOE* alleles. These transgenic rats provide the powerful models for research into ApoE isoform-related human diseases. As the first group to utilize these models in the cardiovascular research, we would like to explore whether human ApoE2 had a positive or protective influence on heart function in rat models, and to reveal whether human ApoE2 could endow enhanced heart contractile function.

In vivo echocardiography, cellular physiological strategies, and molecular assays were used to study the cardiac phenotypes and underlying molecular mechanisms of hApoE KI (hApoE2, hApoE3, and hApoE4) rats in comparison to wide-type (WT) and ApoE knock-out (ApoE KO) rats. We found that hApoE2 rats exhibited enhanced heart function, demonstrated by elevated heart contractile function and stronger stimulated contractility of isolated cardiomyocytes, which might be associated with higher mitochondrial energy generation and elevated intracellular Ca^2+^ transient of hApoE2 cardiomyocytes.

## 2. Materials and Methods

### 2.1. Animal

All animal experiments were approved by the Institutional Animal Care and Use Committee (IACUC) at Georgia State University (protocol code A19054 and date of approval 8 July 2019) and complied with the National Institutes of Health (NIH) Guide for the Care and Use of Laboratory Animals. Rats were purchased from Envigo RMS (Saint Louis, MO, USA, previously named Horizon Discovery and SAGE Labs), housed in a 12-h light/dark cycles environment, and maintained on a standard diet. The male Sprague Dawley WT rats (order code: 002), homozygous HsdSage:SD-*ApoE^em1Sage^* rats (ApoE KO, order code: 350), homozygous hApoE2 KI rats (hApoE2, order code: 394), homozygous hApoE3 KI rats (hApoE3, order code: 395), and homozygous hApoE4 KI rats (hApoE4, order code: 359) were used for experiments. Gender differences were compared between males and females of hApoE2 KI rats. Neonatal rats were generated by breeding the Sprague Dawley WT rats. Adult rats used were aged 6 months unless specifically stated. Rat number used are: 30 each for male WT, male hApoE2, male hApoE3, and male hApoE4; 10 male ApoE KO; and 6 female hApoE2.

### 2.2. In Vivo Echocardiography

Noninvasive echocardiography was performed using Visual sonics Vevo 3100 Imaging System (FUJIFILM VisualSonics, Inc., Toronto, ON, Canada). Rats were anesthetized with 2–3% isoflurane, following the hair shaving with a clipper and Nair hair remover lotion (Church & Dwight Co., Inc., Ewing, NJ, USA) from operation areas in a supine position atop a heating pad with embedded ECG leads. Body temperature was maintained and monitored during the whole process. The ocular gel was applied to both eyes to prevent drying of the sclera. The four paws were given an application of electrode gel and the electrodes were taped on for ECG signals. Preheated ultrasound gel was applied to the chest. 2D Echocardiography Imaging (B-mode) was performed to obtain a clear view of the short-axis left ventricle (LV). M-mode tracings of LV were recorded for the analysis of the heart function parameters, LV ejection fraction (LVEF) and LV fractional shortening (LVFS), and the LV anterior wall thickness during the diastolic phase (LVAWd). The tracings for pulse wave Doppler of mitral annulus velocity were recorded under apical 4-chamber view for the analysis of the diastolic heart function parameter E/A ratio [22]. The heart rate for M-mode recording was maintained at around 400 beats per minute (min). The heart rate for pulse wave Doppler recording was maintained at 300 to 350 beats per min to see the separated E and A waves. The gel was cleared after the recording. When the rats awakened, they were returned to their cages. For data collected <24 weeks, 19 to 27 rats were used per WT and hApoE KI groups, and 6 to 12 rats were used for ApoE KO male group. For data collected ≥24 weeks, 3 to 8 rats were used per ApoE KO male group. Six female hApoE2 rats were used for gender comparison of E/A ratio.

### 2.3. Arterial Strain Measurement by Ultrasound

Arterial strain analysis was performed on the rat’s abdominal aorta using Vevo 3100 Imaging System. Rats were anesthetized with isoflurane, following the hair removal from the abdomen in a supine position. Warmed ultrasound gel was applied, and the probe was positioned to visualize the abdominal aortic segments between the branching of the superior mesenteric artery and the branching of the superior renal arteries. M-mode tracings of aortic crossing view were recorded. Aortic strain, representing the arterial stiffness/elasticity, was calculated using a radial strains formula (Equation (1)):

ε = (L − L_0_)/L_0_, (1)

percent ratio of the systolic-diastolic diameter changes and diastolic diameter of aorta, where ε = strain, L = instantaneous length, and L_0_ = baseline length at the time of measurement [23,24].

### 2.4. Blood Pressure and Heart Rate Measurement

Sedated arterial blood pressure was measured with a microtip catheter inserted into the left common carotid artery. Briefly, rats were anesthetized with a ketamine and xylazine mixture (80:4 mg/kg BW, ip. injection) and placed on a warm pad (37 °C). The left common carotid artery was carefully exposed via a 1 to 1.5-cm midline incision in the ventral neck region. The tip of the artery toward the head was ligated with a suture (5-0 silk), and the tip toward the heart was occluded with a micro clip. A small cut was made in the vessel wall, allowing the insertion of a catheter (PE10 tubing, BD, Franklin Lakes, NJ, USA) containing a sterile 10% heparin solution in saline. Blood was directed to a pressure transducer through the catheter to obtain computerized blood pressure measurements after removing the micro clip. The rats were stabilized, and the mean, systolic, diastolic blood pressure, and heart rates were monitored for at least 30 min in anesthetized status. Pressure–volume signals were digitized at 1 kHz and recorded using ADInstruments LabChart DAQ software (AD Instruments, Hastings, UK). The rats were euthanized after measurement.

Non-invasive arterial pressure was measured using CODA^®^ Noninvasive Blood Pressure System (Kent Scientific Corporation, Torrington, CT, USA) following the manufacturer’s instructions. Briefly, rats were acclimated by placing them in the holders with tail cuffs for 15 min on the warming platform (32 to 35 °C) for 3 consecutive days before the actual recording. Both acclimation and actual recording used a protocol of 20 cycles. The mean, systolic, diastolic blood pressure, and heart rates were monitored with this tail-cuff system in conscious condition. Data was collected on the fourth day and exported as Excel files. The actual experiment consists of at least six blood pressure measurements in each rat.

### 2.5. Heart Weight Measurement

Rats were euthanatized at the age of 6 months with the CO_2_ inhalation method followed by cervical dislocation according to the GSU IACUC Policy. The animal was transcardially perfused with 0.1 M phosphate-buffered saline (PBS, pH 7.4) until the liver was cleared of blood. Later, hearts were dissected and collected. The weights of hearts were recorded and represented with normalization of body weight or tibia bone length.

### 2.6. Histology and Microscopy of Heart Slices

Dissected hearts were fixed in 10% formalin solution for at least 3 days, following standard dehydration processing and paraffin embedding. Heart tissues were cross sectioned at a thickness of 5 µm and stained with the standard hematoxylin and eosin (HE) method, Picrosirius red stain kit (Cat. # 24901, Polysciences, Inc., Warrington, PA, USA), or immunofluorescence (IF) staining. Slices were mounted with Permount mounting medium (P15, Thermo Fisher Scientific, Waltham, MA, USA) for HE and Picrosirius red staining, or ProLong™ Gold Antifade Mountant with DAPI (P36935, Thermo Fisher Scientific) for IF staining. The antibodies used in this study are listed in Supplementary Material Table S1. For IF of paraffin slices, an antigen retrieval step was performed before staining. Slices were imaged by an Olympus BX53 microscope. The ratio of LV wall thickness and LV radius was calculated using images captured in HE-stained cross-sections of the hearts with Image J software (U. S. National Institutes of Health, Bethesda, MD, USA, https://imagej.net/ij/index.html) with equation

(2) $$\sqrt{\left(A_{lv}-A_{C}\right)/\pi }/\sqrt{A_{lv}/\pi },\;$$

*A_lv_*, cross area of LV, *A_C_*, inner chamber area of LV, π, mathematical constant. For CF555-WGA staining, the individual cell size/area of the cross-sectioned cardiomyocytes was quantified from at least 3 captures per rat using Image J software.

### 2.7. Cell Isolation and Culture

Adult rat cardiomyocytes were isolated using a modified protocol described previously [25]. Briefly, a rat at age 6–8 months was overdosed with isoflurane under a fume hood and euthanized by cervical dislocation. The heart was quickly removed into ice-cold Kreb’s solution (118 mM NaCl, 4.7 mM KCl, 1.8 mM CaCl_2_·2H_2_O, 1.2 mM MgSO_4_·7H_2_O, 1.2 mM NaH_2_PO_4_, adjust pH = 7.4, sterile filter). Aorta was intubated, allowing the heart to be perfused in a retrograde manner. The heart was perfused retrogradely with 30 mL of ice-cold Kreb’s solution mixed with 10 U/mL heparin using a syringe pump (AL-300, World Precision Ins., Sarasota, FL, USA) at a speed of 5 mL/mL. Then the heart was perfused with 20 mL of EDTA buffer (130 mM NaCl, 5 mM KCl, 0.5 mM NaH_2_PO_4_, 10 mM HEPES, 10 mM glucose, 10 mM BDM, 10 mM Taurine, 5 mM EDTA, adjust pH = 7.8, sterile filter), 10 mL of perfusion buffer (130 mM NaCl, 5 mM KCl, 0.5 mM NaH_2_PO_4_, 10 mM HEPES, 10 mM glucose, 10 mM BDM, 10 mM Taurine, 1 mM MgCl_2_, adjust pH = 7.4, sterile filter), and pre-warmed 60 mL of collagenase buffer (0.5 mM collagenase II, 0.5 mM collagenase IV, 0.05 mM protease XIV, freshly made with perfusion buffer) at the same speed. After 30 min digestion with enzymes, the heart was removed from the catheter, both atria were cut off, and the pericardium was carefully peeled off with forceps. The ventricle tissue was gently chopped into ~1.5 mm × 1.5 mm pieces in 9 mL of fresh collagenase buffer and digested in a water bath for another 5 to 10 min with occasional mixing using a 5 mL disposable pipette. Tissue suspension was pipetted for a further 2 min after adding 20 mL of stop solution (5% FBS prepared in perfusion buffer). A 100 µm pore-size strainer was used to remove the undigested tissue debris. Cardiomyocytes were separated from other cells by gravity settlement for 20 min. Ca^2+^ was sequentially re-introduced by adding 6 mL of Ca^2+^ buffer [4.5 mL perfusion buffer mixed with 1.5 mL culture medium (0.1% BSA, 1× ITS, 0.1 mM BDM, 1× CD lipid, prepared in M199 medium, protect from light), yielded 0.34 mM Ca^2+^], 3 mL of culture medium (yielded 0.68 mM Ca^2+^), and 9 mL of culture medium (yielded 1.02 mM Ca^2+^) with a 10 min interval. Cells were placed in a 50 mL tube horizontally in a water bath during this process, to avoid overlapping. Finally, the supernatant was discarded after gravity settlement for 10 min, and cardiomyocytes were resuspended in the culture medium (1.26 mM Ca^2+^). Isolated cardiomyocytes were ready for culture with culture medium in a typical cell culture incubator (CO_2_ free) or for other assays.

H9C2 rat cardiomyoblast cells were purchased from ATCC (CRL-1446, ATCC, Manassas, VA, USA). Neonatal rats at 1–3 days old were used for cardiomyocyte isolation using Pierce Primary Cardiomyocyte Isolation Kit (Cat. # 88281, Thermo Fisher Scientific, Waltham, MA, USA) following the manufacturer’s instructions. Cells were cultured in DMEM medium (10-013-CV, Corning Inc., Corning, NY, USA) supplemented with 10% Fetal Bovine Serum (FBS) and 1% penicillin/streptomycin. Human ApoE2, 3, or 4 proteins were expressed in H9C2 cells using the pCMV4-ApoE2, 3, or 4 plasmids (Plasmid # 87085, 87086, 87087, Addgene, Watertown, MA, USA) and Lipofectamine™ 2000 Transfection Reagent (Cat. # 11668019, Thermo Fisher Scientific, Waltham, MA, USA) following the manufacturer’s instructions. After the medium change, cells were continually cultured for 24–48 h and ready for Seahorse and Western blotting analysis.

### 2.8. Staining and Microscopy of Single Cardiomyocytes from Adult Rats

Single cardiomyocytes isolated from adult rat hearts were fixed with 4% formaldehyde for 15 min at 37 °C, followed by 3 washes with phosphate-buffered solution (PBS). For mitochondria staining, cells were permeabilized with 0.2% Triton X-100 for 10 min, protein blocked (HK112-9K, BioGenex, Fremont, CA, USA) for 20 min, and incubated in anti-ATP5A1 antibody overnight at 4 °C, followed with specific secondary antibody (Supplementary Material Table S1). For F-actin staining, cells were stained using DyLight^TM^ 554 Phalloidin for 15 min at room temperature. After two washes with PBS, cells were mounted with ProLong™ Gold Antifade Mountant with DAPI. The images were captured by Carl Zeiss LSM 800 confocal microscope.

### 2.9. Seahorse XF Assays

The XFe96 Seahorse Extracellular Flux Analyzer (Bio-Rad Laboratories, Hercules, CA, USA) was used to measure oxygen consumption rate (OCR) in rat cardiomyocytes. H9C2 cells (40,000 per well) or primary neonatal rat cardiomyocytes (40,000 per well) were plated in FXe 96 well cell culture microplates. One hour before the assay, cells were incubated with XFe assay medium containing 25 mM glucose, 10 mM Na^+^ pyruvate, and 2 mM glutamine with pH 7.42 in a non-CO_2_ incubator for 40 min before placing it in the Seahorse Extracellular Flux Analyzer. Oxygen consumption rates were automatically measured at base level and after sequential injections of oligomycin (port A, 2.5 µM), carbonyl cyanide-p-trifluoromethoxyphenylhydrazone (port B, FCCP, 2 µM), and antimycin A plus rotenone (port C, AA/Rot, 2/4 µM) in 3 cycles, and each cycle contains 3 min mixing, 0 min waiting, 3 min measuring periods. In case of adult rat cardiomyocytes (15,000 per well), plates were pre-coated with 5 µg/mL laminin, prepared in PBS for at least 1 h at 37 °C, and cells were cultured overnight after isolation. One hour before the assay, culture medium was replaced by FXe medium containing 5 mM glucose, 1 mM Na^+^ pyruvate, and 4 mM glutamine supplemented with CD lipid concentrate (Cat. # 11905031, Thermo Fisher Scientific). Oxygen consumption rates (OCR) were automatically measured at base level [4× (1 min mix, 1 min wait, 2 min measure)] and after sequential injections of oligomycin (2 µM) [3× (0.5 min mix, 0 min wait, 2 min measure)], FCCP (4 µM) [3× (1 min mix, 2 min wait, 2 min measure)], and AA/Rot (2/4 µM) [5× (1 min mix, 1 min wait, 2 min measure)]. Data were normalized by protein concentration measured using BCA assay, and consisted of 17–45 well repeats per group from 4 independent experiments.

### 2.10. Cardiomyocyte Contractility and Intracellular Ca^2+^ Signaling

For the assessment of cardiomyocyte shortening and intracellular Ca^2+^ transients, freshly isolated cardiomyocytes from adult rats at age 6–8 months were loaded into the cell-stimulation chamber of Myocyte Ca^2+^ and Contractility Systems (IonOptix, Westwood, MA, USA) in loading buffer (137 mM NaCl, 5.4 mM KCl, 1.3 mM CaCl_2_, 0.5 mM MgCl_2_, 10 mM HEPES, 5.5 mM glucose, and 0.5 mM probenecid, adjust pH = 7.4, warmed to 37 °C before use). Cells were stained with Fura 2-AM (ab120873, Abcam, Cambridge, UK) at a final concentration of 1 µM for 15 min at room temperature, following two washes with loading buffer. Fura 2-AM signal representing the intracellular Ca^2+^ concentration was recorded as the ratio of fluorescence intensity excited at 340 nm and 380 nm in the dark by electric stimulation at a given frequency (1 Hz at 5–9 volts), with simultaneous sarcomere length detection for shortening/re-lengthening. Data was recorded with IonWizand 7 (IonOptix) and analyzed following the manufacturer’s instructions. At least 13 cells from 3 rats were used for data analysis per group.

### 2.11. Western Blotting Assays

Briefly, 25 µg of protein from the rat heart lysate (rat aged 6 months) or cell lysate (n = 3–6 rats/group) was denatured and loaded to gradient tris-glycine SDS-PAGE gel. Protein lysates of rat hearts were from 6 rats per group, and the bar graphs were made with the data collected from at least 4 independent assays. Proteins were transferred onto nitrocellulose or PVDF membranes and blocked with 5% w/v non-fat milk prepared in TBS solution. Membranes were probed with specific antibodies, then incubated with HRP-linked secondary antibody (anti-mouse or anti-rabbit IgG). Supplementary material Table S1 summarized the antibodies used. Protein bands were visualized using SuperSignal™ West Pico PLUS Chemiluminescent substrate (Cat. # 34577, Thermo Fisher Scientific, Waltham, MA, USA). Images were captured with Amersham™ Imager 680 Western blotting imaging system (BIOKÉ, Leiden, The Netherlands). The band intensity was quantified by ImageJ software.

### 2.12. Data Analysis

All data were expressed as the mean and standard error of the mean (SEM). Prism (GraphPad Software Inc., San Diego, CA, USA) was used for statistical analyses. A one-way ANOVA followed by a Tukey post hoc test for multiple comparison, or student *t*-test for comparison between two groups, were used. Differences with *p* < 0.05 were considered significant.

## 3. Results

### 3.1. hApoE2 Rats Show Enhanced Left Ventricular Function In Vivo

**Figure 1:**
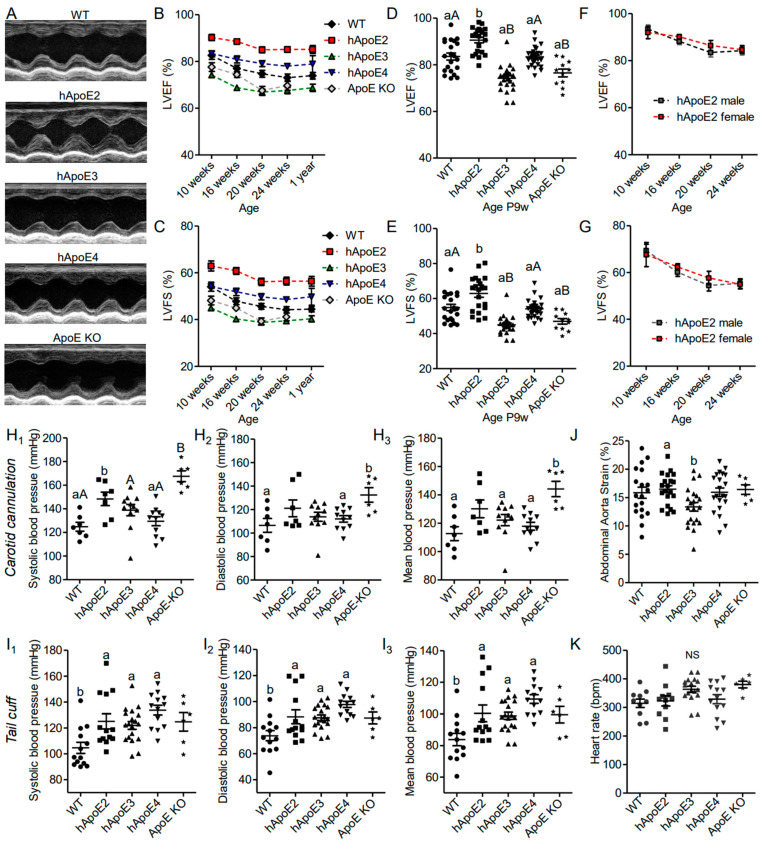
hApoE2 rats showed enhanced heart function, which influenced the diastolic blood pressure of the carotid artery but not distant tail blood pressure. (**A**) Heart function of different rats was measured and compared by M-mode echocardiography. (**B**–**E**). Left ventricular ejection fraction (LVEF, (**B**,**D**)) and left ventricular fractional shortening (LVFS, (**C**,**E**)) measured by echocardiography were compared between different male rats at different ages. (**F**,**G**). LVEF and LVFS of male and female hApoE2 rats were compared. (**H**). Blood pressure was detected through arterial cannulation of the left carotid artery in rats under anesthesia. (**I**). Blood pressure measured with a tail-cuff technique (CODA^®^ Noninvasive Blood Pressure System) was compared between awake rats. (**J**). Arterial strain measured in the abdominal aorta of different rats was compared. (**K**). Heart rate of different rats under awake condition. One-way ANOVA followed by a Tukey post hoc test. a vs. b, A vs. B, *p* < 0.05, NS, no significant difference. Rat number was indicated by symbols in the figures.

First, we assessed the cardiac performance of the male hApoE, WT, and ApoE KO rats at different ages using echocardiography, and found that hApoE2 rats exhibited enhanced LVEF and LVFS compared to other groups (Figure 1A–E). hApoE3 and ApoE KO rats showed reduced LVEF and LVFS compared to those of WT and hApoE4 rats. hApoE4 rats showed similar LVEF and LVFS as WT rats. Male and female hApoE2 rats did not show a significant difference in LVEF and LVFS (Figure 1F,G). Furthermore, we investigated blood pressure via cannulation into the left common carotid artery and found that the systolic blood pressure of hApoE2 was slightly increased compared to these of WT, hApoE3, and hApoE4 rats (Figure 1H). ApoE KO rats exhibited significant increase in blood pressure compared to WT, hApoE3, and hApoE4 rats. Tail-cuff arterial pressure, which usually underestimates the central arterial pressure [26], did not show a significant difference among hApoE KI rats. (Figure 1I). All transgenic rats showed slightly higher tail-cuff readings compared to WT rats. In our previous study [27], we found that hApoE2 rats developed spontaneous hyperlipidemia without plaque formation at age 6 months. The high lipids levels in the vessels might cause vascular problems, such as increased stiffness/decreased elasticity of arterial walls. Stiff arteries usually result in higher systolic blood pressure because of the reduced capacity and lower diastolic pressure, which occurs because of less elastic recoil to support the diastolic pressure. Therefore, arterial strain was measured by ultrasound-based estimation at the abdominal aorta. A decreased strain value usually indicates increased arterial stiffness. hApoE2 rats did not exhibit reduced arterial strain values, indicating intact arterial stiffness/elasticity (Figure 1J). The heart rate measured under conscious conditions was not influenced by ApoE isoforms, either (Figure 1K). These data indicate that the increased systolic blood pressure of hApoE2 rats may be the result of the enhanced LV contractile function.

### 3.2. hApoE2 Rats Do Not Exhibit Detrimental Heart Remodeling

**Figure 2:**
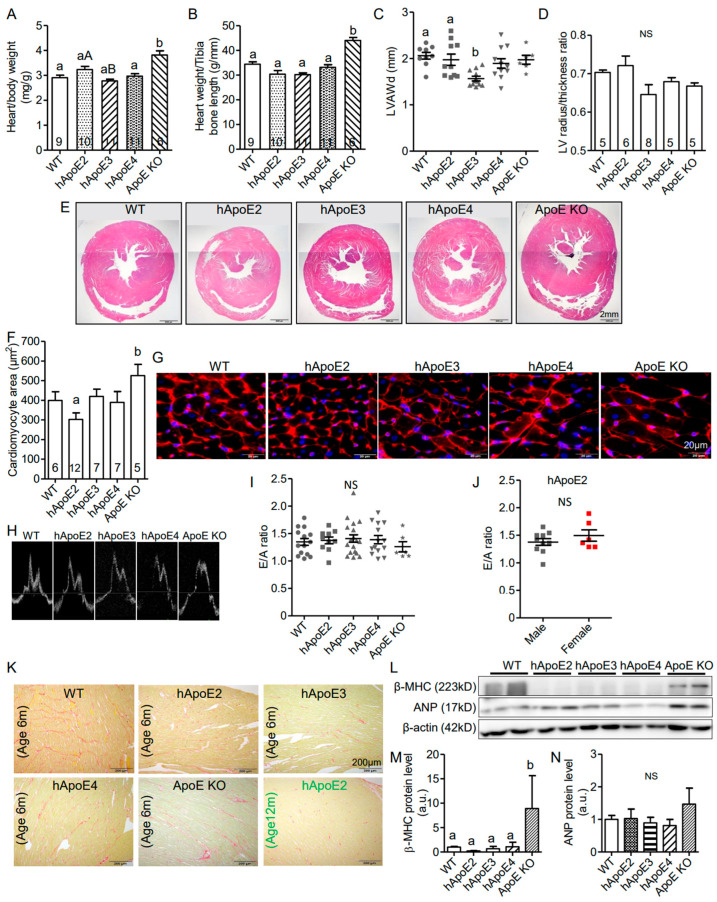
Multiple tests demonstrated that hApoE2 rats did not exhibit detrimental heart remodeling. (**A**,**B**) Heart weight normalized by body weight (**A**) and tibia bone length (**B**) were compared between different rats. (**C**) Left ventricular end-diastolic anterior wall thickness (LVAWd) measured at postnatal age 6 months were compared. (**D**) Left ventricular heart wall thickness represented by the ratio of the left ventricular radius and wall thickness was compared via analysis of the Hematoxylin and eosin (HE)-stained cross-sections of the hearts. (**E**) Representative HE-stained cross-sections of the rat hearts were shown. (**F**) Average cell area of cardiomyocytes from cross-sectioned rat hearts was compared. (**G**). Cell membranes of cross-sectioned rat cardiomyocytes were stained by CF555 wheat germ agglutinin (WGA) conjugates in red. Nuclei were stained by DAPI in blue. (**H**) Representative tracings for pulse wave Doppler for the rat mitral annulus velocity were shown. E and A wave ratio, as a marker of the left ventricular diastolic function and stiffness, were compared in different male rats at postnatal 6 months (**I**) and between male and female hApoE2 rats at postnatal 6 months (**J**). (**K**) Images of Picrosirius red staining were used to appraise collagen networks in rat left ventricles. Collagen fibers were stained in red with standard light microscopy. Muscle fibers were stained yellow. (**L**) Western blotting showed the expression levels of β-myosin heavy chain (anti-β-MHC) and atrial natriuretic peptide (anti-ANP) as cardiac lesion markers. (**M**,**N**) Protein expression levels of β-MHC and ANP were quantified and compared in the heart lysate of different rats. β-actin was used as a loading control. One-way ANOVA followed by a Tukey post hoc test for comparison among different male rat groups; student *t*-test for comparison between male and female hApoE2 rats. a vs. b, A vs. B, *p* < 0.05, NS, no significant difference. Rats number used for statistical analysis was labeled in bar graphs or indicated by symbols in scatter plots. Five rats per group were used for Western blotting analysis.

hApoE2 rats have smaller body size, as well as the absolute heart weight, compared to WT, hApoE3, hApoE4, and ApoE KO rats (Supplementary Figure S1). However, the heart size of hApoE2 rats normalized to body weight or tibia bone length was found to be similar to those of WT and hApoE4 rats, and relatively larger than those of hApoE3 rats (Figure 2A,B). LV wall thickness was not seen in echocardiography (Figure 2C) or histological HE-stained slices (Figure 2D,E) of hApoE2 hearts. Surprisingly, we observed that the individual cell size of hApoE2 cardiomyocytes was slightly, but not statistically significantly, smaller than that of WT, hApoE3, and hApoE4 cells (Figure 2F,G). ApoE KO rats showed both increased heart weight (Figure 2A,B, Supplementary Figure S1B) and larger cell size (Figure 2F,G), suggesting the hypertrophic changes in their LV.

The pulse wave Doppler of mitral valve leaflet tips was recorded to analyze the diastolic heart function. The E wave corresponds to the mitral inflow velocity pattern during the early diastolic filling of the LV, and the A wave corresponds to the mitral inflow velocity pattern during the late diastolic filling of the LV. The E/A ratio is a parameter to evaluate the diastolic function of the LV. A reduced E/A ratio is a sensitive early sign suggesting a stiffened myocardium. However, reduced E/A ratio was not seen in hApoE2 rats (Figure 2H–J). ApoE KO rats showed a reduced trend of E/A ratio (Figure 2I) similar to that was found in ApoE KO mice [28].

Myocardial fibrosis is usually observed at the relatively later stage of heart failure. We used Picrosirius red staining to visualize the fibrotic tissue. Increased myocardial fibrosis was not observed in hApoE2 heart slices at 6 months age or 12 months age (Figure 2K).

**Figure 3:**
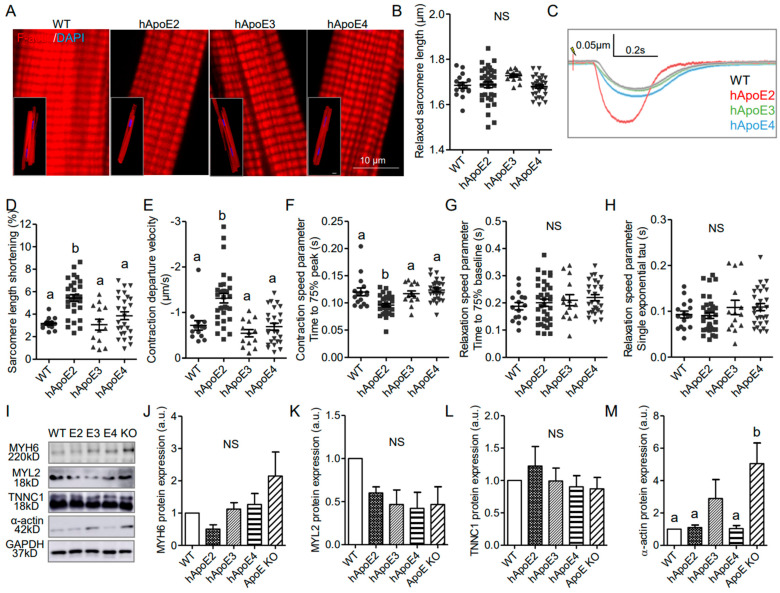
Isolated adult hApoE2 cardiomyocytes showed stronger cellular contractility, which is not due to the mechanisms regulating actin and myosin filament assembly. (**A**) DyLight^TM^ 554 Phalloidin stained the F-actin labeling the thin filaments (red) of the cardiomyocytes isolated from adult rats. Nuclei were stained by DAPI in blue. (**B**) The relaxed sarcomere length of isolated adult cardiomyocytes was compared. (**C**) Representative traces of sarcomeric contractions are shown. Isolated cardiomyocytes were stimulated by 5 to 8 volts pacing stress at 1 Hz. (**D**) Percent change of sarcomere length during the contraction was compared between cell groups. (**E**) The departure velocity, a value indicating the maximal rate of change during the contraction, was compared. (**F**) The time to 75% of maximal departure velocity indicating how fast contraction happened was compared. (**G**) The time to 75% baseline, representing the time that sarcomere restored 75% of length change between the shortest length after stimulation, and initial baseline length were compared (noting that the sarcomere may not back to the baseline length during the relaxation phase). (**H**) The single exponential tau, the exponential decay time constant of the function, as another means of characterizing the speed of relaxation, was compared as well. A greater tau value means a longer relaxation time. (**I**–**M**) Western blotting was applied to detect the expression levels of cardiac myosin heavy chain (anti-MYH6), myosin light chain (anti-MYL2), troponin C (anti-TNNC1), and the thin filament protein α-actin in heart lysate of different adult rats. Glyceraldehyde 3-phosphate dehydrogenase (anti-GAPDH) was used as a loading control. One-way ANOVA followed by a Tukey post hoc test. a vs. b, *p* < 0.05, NS, no significant difference. Cells indicated by symbols were from three rats per group. Three to five rats per group were used for Western blotting analysis.

We further detected heart lesion markers, β–myocin heavy chain (β-MHC), and atrial natriuretic peptide (ANP) (Figure 2L–N), as well as hypertrophy marker α-actin (Figure 3I,M), in the heart lysate of rats via Western blotting. Only ApoE KO heart lysate showed significantly increased expression of β-MHC (Figure 2M) and α-actin (Figure 3M). hApoE2 rats showed no significant difference in the expression of β-MHC, ANP, or α-actin in heart lysate compared to WT rats. In summary, hApoE2 rats did not exhibit detrimental heart remodeling.

### 3.3. Isolated Adult hApoE2 Cardiomyocytes Show Stronger Cellular Contractility

Cardiomyocytes are the individual functional units of cardiac muscle providing the contractile power of the heart. A sarcomere is the basic contractile unit of muscle fiber. To investigate whether human ApoE isoforms influence the contractility of the cardiomyocytes, cardiomyocytes were isolated from adult hApoE2, WT, hApoE3, and hApoE4 rats at age 7–8 months (ApoE KO rats were not included due to the availability) and tested for different parameters, such as sarcomere histology, length, and contractility function. The sarcomeric histology of hApoE KI cardiomyocytes did not show a significant difference (Figure 3A). Sarcomere length of all four cell groups was similar in the resting state (Figure 3B). When electrically stimulated at 1 Hz, hApoE2 cells showed more percent change of sarcomere length (Figure 3D) and faster contractile speed (Figure 3E,F), but no changes in relaxation speed (Figure 3G,H), compared to other groups. The main contractile elements forming sarcomeres are thin and thick filaments, majorly composed of α-actin and myosin, respectively [29]. Troponin (T, I, and C) sits along the actin strands, regulating the contractile process, in which troponin C binds to Ca^2+^ ions initiating the conformational change in tropomyosin, facilitating the binding between actin and myosin. Thus, we detected the protein levels of cardiac myosin heavy chain, myosin light chain, α-actin, and troponin C, and found no significant difference among the heart lysates of WT and three hApoE KI rats (Figure 3I–M). ApoE KO heart lysate exhibited a significantly increased protein level of α-actin (Figure 3I,M), a sign of hypertrophic ventricle [30]. Overall, the changes of contractile filaments of sarcomeres are not seen in hApoE2 cardiomyocytes.

### 3.4. ApoE2 Expressing Cardiomyocytes Present Enhanced Fatty Acid β-Oxidation

**Figure 4:**
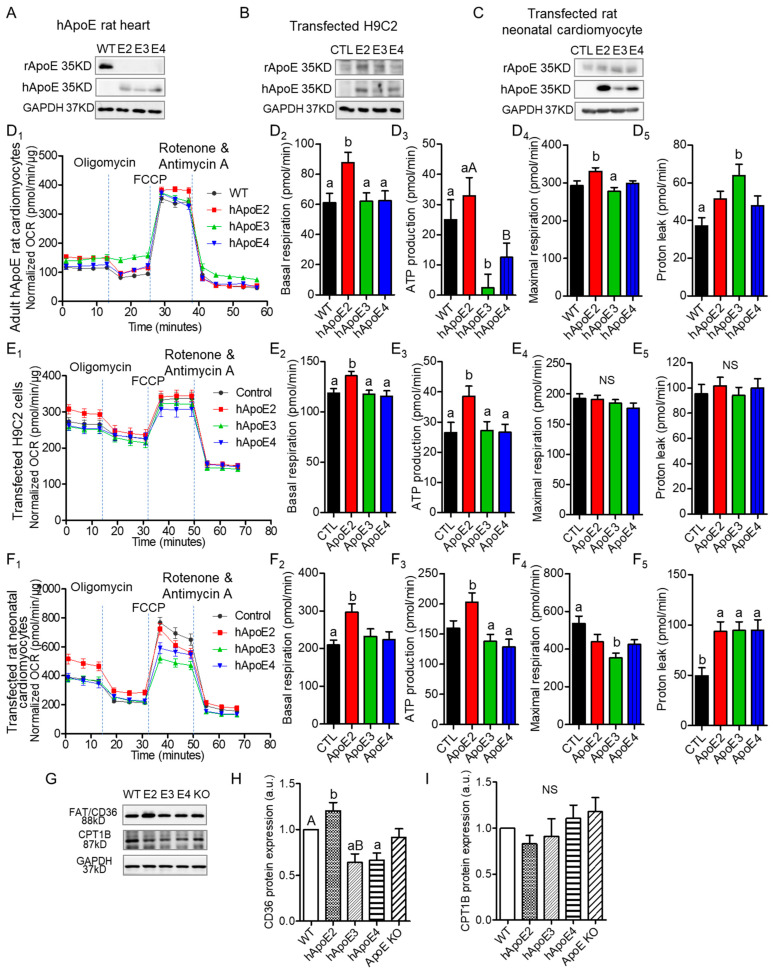
ApoE2 expressing cardiomyocytes exhibited elevated energy production through mitochondrial respiration/fatty acid β-oxidation. Western blotting detected the protein expression levels of rat ApoE (anti-rApoE) and human ApoE (anti-hApoE) in heart lysate of adult rats (**A**), H9C2 cells transfected with different human ApoE isoforms (**B**), and rat neonatal cardiomyocytes transfected with different human ApoE isoforms (**C**). Seahorse XF assays, a method to detect the mitochondrial function, were applied to measure the oxygen consumption rate (OCR) using isolated cardiomyocytes of adult rats at age 7–8 months (**D**), transfected H9C2 cells (**E**), and transfected rat neonatal cardiomyocytes (**F**). Representative Seahorse XF assay traces, basal OCR, ATP production rate, maximal OCR, and proton leak were compared between groups of cells. Seahorse data were normalized with total cellular protein content using BCA assays. Data were summary of 17–45 reads from 4 independent assays for each group. (**G**–**I**) The expression levels of fatty acid transporting proteins (anti-FAT/CD36) on cell membrane and carnitine palmitoyltransferase 1 (anti-CPT1) on the outer membrane of mitochondria, detected with Western blotting in heart lysate of adult rats and compared between rat groups. One-way ANOVA followed by a Tukey post hoc test. a vs. b, A vs. B, *p* < 0.05, NS, no significant difference. Four individual rats/experiments were performed for each Seahorse assays. Four to six rats per group were used for Western blotting analysis.

Oxidation of fatty acids is responsible for over 90% of the energy requirements and is necessary for optimal cardiac contractile. β-oxidation of fatty acids occurs primarily in the mitochondria. Therefore, we investigated mitochondrial function of cardiomyocytes expressing different ApoEs via Seahorse XF assays (Figure 4A–F). We used primary cardiomyocytes isolated from adult rats at age 7–8 months (Figure 4A), H9C2 cells transfected with different human ApoE isoforms (Figure 4B), and rat neonatal cardiomyocytes transfected with different human ApoE isoforms (Figure 4C). Oxygen consumption rate (OCR) could reflect the basal β-oxidation rate and optimized β-oxidation capability. OCR results indicated that isolated cardiomyocytes from adult hApoE2 rats exhibited higher basal respiration and ATP-linked respiration (ATP production) than those of hApoE3 and hApoE4 cells (Figure 4D_1–3_), indicating a higher basal energy generation rate of hApoE2 mitochondria. There was a correlation between the ATP-linked respiration of these rats and their heart performance detected through echocardiography: hApoE2 groups showed an increasing trend, while hApoE3 groups showed a decreasing trend. hApoE2 cardiomyocytes showed higher maximal respiration as well, especially compared to that of hApoE3 cells (Figure 4D_4_), indicating hApoE2 cardiomyocytes may have robust ability to maximize the mitochondrial β-oxidation. Proton leak represents the proton amount that freely moves across the mitochondrial membrane during unit time. If the amount is high, mitochondrial respiration will not occur due to the loss of respiration-dependent proton gradient [31]. hApoE3 cardiomyocytes showed a higher proton leak than that of WT cells (Figure 4D_5_), indicating the dissipated membrane potential and reduced efficiency of β-oxidation in hApoE3 cardiomyocytes. Human ApoE2-overexpressed H9C2 cells and neonatal cardiomyocytes from WT rats exhibited similar increased basal respiration and ATP-linked respiration, but neither the maximal respiration nor the proton leak (Figure 4E,F), indicating human ApoE2 endowed the ability of cardiomyocytes to produce more ATP in basal condition. In neonatal cardiomyocytes, human ApoE3 overexpression reduced the maximal respiration compared to the control group (Figure 4F_4_). These data suggest that a correlation between mitochondrial β-oxidation of adult cardiomyocytes from WT, hApoE KI, and ApoE KO rats and their cardiac function and myocyte contractility. The higher energy production via fatty acid oxidation conferred by human ApoE2 might be one mechanism contributing to the potent cardiac contractility of rats.

Cardiac health and energy generation are directly tied to mitochondrial mass and dynamics [32,33,34,35]. Expression of mitochondrial proteins, including COX IV, Hsp60, VDAC1, Tim23, and Tom20, were measured, but only Tom20 of the hApoE2 heart lysate showed slightly higher expression than that of the hApoE4 group (Supplementary Figure S2A–F). As the primary energy converter and highly dynamic organelles, mitochondria constantly undergo fission and fusion events, referring to “mitochondrial dynamics”, which requires multiple factors [36,37,38]. We detected factors involved in mitochondrial fusion (Opa1, Mfn1, and Mfn2) and fission (MFF, Fis1, and Drp1), but no significant difference was identified among hApoE KI heart samples (Supplementary Figure S2G-M). IF staining with anti-mitochondrial protein ATP5A antibody did not show histologic difference among cardiomyocytes from adult rats carrying different human ApoE isoforms (Supplementary Figure S2N). These data suggest that ApoE isoforms do not influence mitochondrial mass and dynamics.

Fatty acids primarily enter cardiomyocytes via fatty acid protein transporters, such as FAT/CD36, FABPpm, and FATP1 [39]. Previously, we found that hApoE2 rats exhibited moderately increased blood triglyceride [27]. Hence, we detected protein expression of FAT/CD36 in the heart lysate of WT, hApoE KI, and ApoE KO rats, and found that the hApoE2 heart expressed a higher level of CD36 protein than other heart samples (Figure 4G,H). Upon transport to the cytosol, the fatty acid is added to a CoA group by long-chain acyl-CoA synthetase (ACSL). Carnitine palmitoyltransferase 1 (CPT1) on the outer membrane of mitochondria converts fatty acyl-CoA to acylcarnitine, which is transported across the mitochondrial membranes by voltage-dependent anion channels (VDAC) and carnitine translocase (CAT) [39,40]. CPT1 is the rate-limiting enzyme of β-oxidation [41]. Later, CPT2 converts the fatty acylcarnitine back to fatty acyl-CoA, which then enters β-oxidation for acetyl-CoA production. We detected the expression of the CPT1 protein, but no significant difference was identified among the heart lysate of WT, hApoE KI, and ApoE KO rats (Figure 4G,I). These data suggest that hApoE2 cardiomyocytes might have optimized fatty acids availability and transportation across cell membrane, facilitating the oxidation of mitochondrial fatty acids.

### 3.5. Potent Calcium Signal in hApoE2 Cardiomyocytes

**Figure 5:**
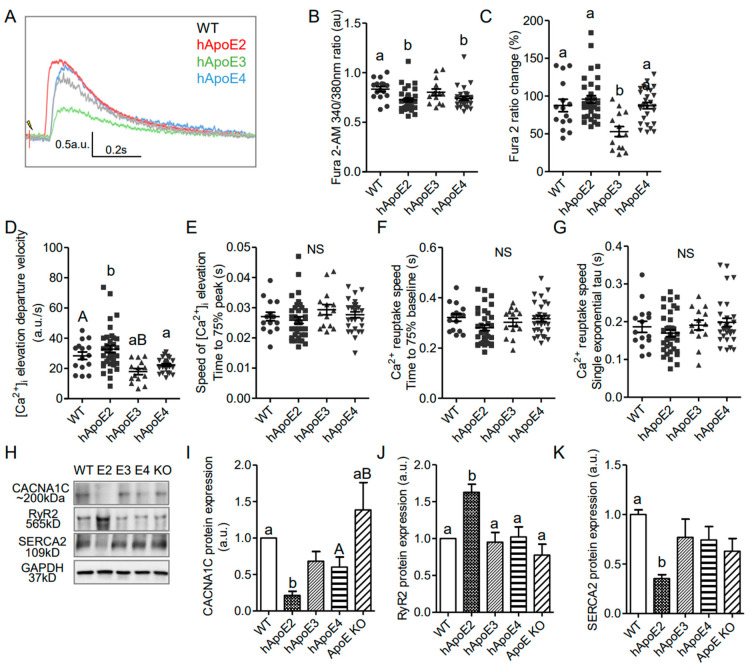
Intracellular Ca^2+^ transients indicated that Ca^2+^ increased more and more quickly after stimulation in isolated adult hApoE2 cardiomyocytes. (**A**) Representative traces of intracellular Ca^2+^ transients are shown. Fura2-AM signal was used as an index of intracellular Ca^2+^ concentration. (**B**) The basal concentration of intracellular Ca^2+^ indicated by the Fura2-AM signal was compared in isolated relaxed adult cardiomyocytes. (**C**) Percent change of Fura2-AM signal was compared in rat cardiomyocytes after electric stimulation of 5 to 8 volts at 1 Hz. (**D**) The departure velocity, indicating the maximal rate of Ca^2+^ change, was compared. (**E**) The time to 75% of maximal departure velocity, indicating how fast the signal increased, was compared. (**F**) The time to 75% baseline, representing the time that the Ca^2+^ signal dropped 75% of total change, a parameter of Ca^2+^ reuptake speed, was compared during relaxation. (**G**) The single exponential tau, equaling the time taken for switching on Ca^2+^ reuptake during the relaxation phase, was compared as well. A greater tau value means a longer Ca^2+^ reuptake time. (**H**–**K**) Western blotting was applied to detect the proteins responsible for intracellular Ca^2+^ signal, using antibodies specific to L-type Ca^2+^ channels (anti-CACNA1C), ryanodine receptors (anti-RyR2), and sarcoplasmic reticulum Ca^2+^ ATPase (anti-ATP2A/SERCA2) in heart lysate of different rats. One-way ANOVA followed by a Tukey post hoc test. a vs. b, A vs. B, *p* < 0.05, NS, no significant difference. Cells indicated by symbols were from three rats per group. Six rats per group were used for Western blotting analysis.

In striated muscle, the initial influx of Ca^2+^ through L-type Ca^2+^ channels on the plasma membrane during the second phase of the action potential is not enough to trigger the contraction of the myofibrils [42]. The ryanodine receptors (RyR), a family of Ca^2+^ release channels on the sarcoplasmic reticulum (SR), represent the primary pathway for Ca^2+^ release during the excitation–contraction coupling process [43]. Then Ca^2+^ is sequestered back into the SR by sarcoplasmic reticulum Ca^2+^ ATPase (SERCA2). To explore whether the mechanism of Ca^2+^-induced Ca^2+^ release (CICR) is involved in the strengthened contractility of hApoE2 cardiomyocytes, we investigated the intracellular Ca^2+^ transients by using the isolated cardiomyocytes from 6- to 8-month rats, and the expressions of the involved channels, by using the heart protein lysate from 6-month rats (Figure 5). Intracellular Ca^2+^ was labeled by Fura2-AM. The fluorescence signal detected in hApoE2 and hApoE4 cardiomyocytes was significantly lower than than in WT cardiomyocytes under resting status (Figure 5B), indicating the lower basal Ca^2+^ level in hApoE2 and hApoE4 cardiomyocytes. The increase in Fura2-AM signal after electric stimulation in hApoE3 cardiomyocytes was significantly smaller compared to other groups, especially the hApoE2 group (Figure 5C), indicating a lower intracellular Ca^2+^ release in hApoE3 cardiomyocytes during the contraction. The speed of Ca^2+^ release was indicated by departure velocity, and was significantly quicker in the hApoE2 group compared to hApoE3 and hApoE4 groups, while hApoE3 cardiomyocytes showed a slower Ca^2+^ release speed than WT and hApoE2 groups (Figure 5D). It took similar time for the intracellular Ca^2+^ to reach the peak levels among different cell groups (Figure 5E). Ca^2+^ was sequestered at a similar speed in all cell groups (Figure 5F,G). When detecting the channels involved in Ca^2+^ release and reuptake, we found the levels of RyR (cardiac isoform 2) [43], responsible for primary Ca^2+^ release from SR during contraction, significantly increased in the hApoE2 group (Figure 5H,J). A decrease in L-type Ca^2+^ channels (CACNA1C) was found in the hApoE2 group compared to WT and ApoE KO groups (Figure 5I). Indeed, an increased expression of CACNA1C was identified in ApoE KO heart lysate compared with hApoE2 and hApoE4 groups (Figure 5I), confirming the hypertrophic change of ApoE KO heart. The SERCA2 responsible for Ca^2+^ reuptake had a lower expression level in hApoE2 heart lysate (Figure 5H,K), but did not show slower Ca^2+^ reuptake in hApoE2 cardiomyocytes during relaxation (Figure 5F,G). These data suggest that hApoE2 cardiomyocytes possess a mechanism allowing quicker and more massive Ca^2+^ release after cell depolarization.

## 4. Discussion

The present study demonstrated the enhanced heart contractile function of hApoE2 rats in comparison to WT, hApoE3, hApoE4, and ApoE KO rats. The enhanced cardiac contractility in hApoE2 rats was not caused by impaired relaxation and stiffened myocardium. We also have presented that rat hApoE2 cardiomyocytes showed stronger and faster sarcomere contractility. Further studies demonstrated that ApoE2-expressed cardiomyocytes had higher ATP generation via fatty acid β-oxidation and elevated intracellular Ca^2+^ signaling after stimulation. Thus, our results unveil that the enhanced heart contractile function of hApoE2 rats might be due to three mechanisms: the dense contractile filaments as the required hardware, and the sufficient energy supply as the driving force, as well as adequate intracellular Ca^2+^ as the assistant secondary message.

As previously reported [27], hApoE2 rats developed spontaneous dyslipidemia. For serum total cholesterol (TC), WT (86.4 ± 5.4 mg/dL), hApoE2 (375.8 ± 17.8 mg/dL), hAPoE3 (86.9 ± 5.2 mg/dL), hApoE4 (85.1 ± 6.6 mg/dL), and ApoE KO (482.0 ± 31.8 mg/dl); For serum total triglyceride (TG), WT (136.5 ± 14.6 mg/dL), hApoE2 (434.1 ± 29.1 mg/dL), hAPoE3 (151.2 ± 7.2 mg/dL), hApoE4 (205.5 ± 14.3 mg/dL), and ApoE KO (187.9 ± 17.2 mg/dL). Following this pathological hyperlipidemia stress, the heart can develop adaptive cardiac hypertrophy, which, in turn, shows an improved contractile force to meet the new body’s demands. When the stress is prolonged, cardiac hypertrophy can lead to heart failure. In this case, the higher LVEF and LVFS could be the consequences of detrimental cardiac remodeling seen at the early stage of heart failure with preserved ejection fraction (HFpEF), a condition presenting with impaired relaxation and stiffened myocardium. HFpEF hearts suffer from hypertrophic changes, increased ventricular stiffness, and cardiac fibrosis. Elevated expression levels of heart lesion markers, such as β-MHC and ANP [44,45], are common in HFpEF as well. However, these detrimental changes were not observed in hApoE2 hearts. Moreover, increased ventricular stiffness and fibrosis that are common in HFpEF were not seen in hApoE2 hearts, either, whereas ApoE KO rats exhibited some phenotypes of HFpEF, similar to those identified in ApoE KO mice [28,46].

In current study, we isolated adult cardiomyocytes from different groups and detected the cardiomyocyte contractility in vitro, which was free of the complicated hormonal and metabolic environment in vivo, and the different contractility among various groups was predominantly caused by the expression of different hApoE isoforms. The results suggest that the stronger contraction of cardiomyocytes is mainly due to the expression of ApoE2 in the heart. Since liver tissue expressed high levels of ApoE [27], we could not exclude the potential effect of liver-expressed ApoE2 on the function of cardiomyocytes.

**Figure 6:**
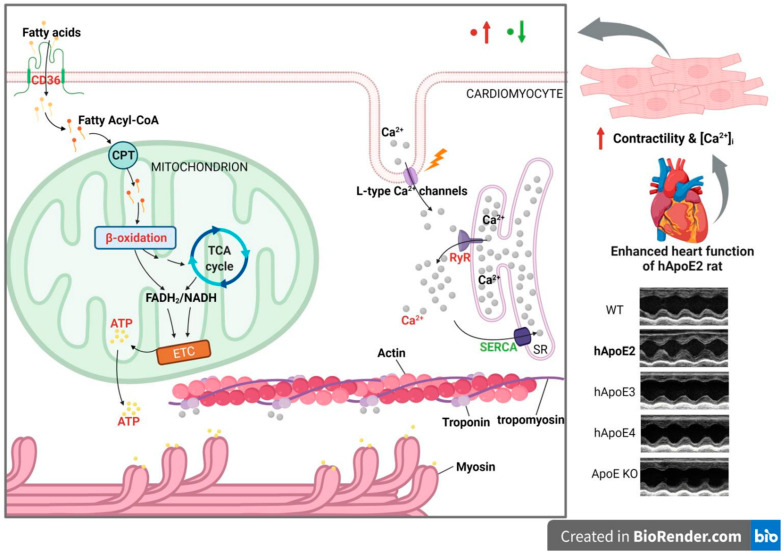
The possible mechanism of how ApoE2 is responsible for enhanced heart function. hApoE2 rat shows enhanced left ventricular function in comparison to hApoE3, E4, WT, and ApoE KO rats via echocardiography. Isolated adult hApoE2 cardiomyocytes showed stronger cellular contractility and higher intracellular Ca^2+^ concentration after electric stimulation. Two mechanisms might contribute to the potent contractility of hApoE2 cardiomyocytes: sufficient energy supply and potent Ca^2+^-induced Ca^2+^ release from the sarcoplasmic reticulum (SR). The former was a result of robust mitochondrial fatty acid β-oxidation and enhanced fatty acid uptake by fatty acid transporting protein CD36. The latter was a consequence of the considerable Ca^2+^ release from the sarcoplasmic reticulum through increased ryanodine receptors (RyR) accompanied by the slightly decreased expression of Ca^2+^ reuptake protein sarcoplasmic reticulum Ca^2+^ ATPase (SERCA). The red-colored objects denote those augmented signals in hApoE2 cardiomyocytes. The green-colored objects denote the suppressed signals. (Created with BioRender.com).

In this study, we found the profuse mitochondrial ATP production and considerable CICR, but not the increased density of contractile filaments in hApoE2 cardiomyocytes. ApoE2 endowed mitochondria with the capability to produce more ATP through fatty acid β-oxidation without affecting the mitochondria quantity in a cell. Elevated CD36 in hApoE2 cardiomyocytes may enhance fatty acid uptake and facilitate fatty acid β-oxidation. However, the CPT1, the key rate-limiting enzyme of β-oxidation, did not change its expression level in hApoE2 cardiomyocytes compared to other ApoE isoforms (Figure 6).

In the present study, we detected several potential intracellular Ca^2+^ signaling modulators associated with cardiomyocyte contractility, including L-type Ca^2+^ channels, RyR2, and SERCA2 (Figure 6). The SERCA2 mediates Ca^2+^ reuptake into SR and thereby promotes cardiomyocyte relaxation, whereas RyR2 mediates Ca^2+^ release from SR and triggers contraction [47]. The balance between SR Ca^2+^ uptake and release is crucial for cardiomyocyte contractibility, mitochondrial function, and cardiac function [48]. We demonstrated that SERCA2 expression in hApoE2 cardiomyocytes was lower than that of WT cardiomyocytes. However, this did not affect the speed of Ca^2+^ reuptake in hApoE2 cardiomyocytes. Furthermore, reduced SERCA2 expression and slower uptake of intracellular Ca^2+^ into SR would not be good for the cardiomyocytes [49]. Importantly, the RyR2 protein levels was significantly higher in hApoE2 cardiomyocytes than those in WT, hApoE3, hApoE4, and ApoE KO cardiomyocytes. These results imply that the major contributor for the increase in Ca^2+^ transient in hApoE2 rats is RYR2. In hApoE2 cardiomyocytes, increased Ca^2+^ transient after stimulation exposes more active sites on the actin, and elevated ATP level increases the number of cross-bridge formations between myosin and actin. These two factors strengthen the power stroke of each sarcomere contraction of hApoE2 cardiomyocytes compared to the cardiomyocytes expressing other ApoEs.

Whether and how the ApoE2 allele is associated with susceptibility to CHD remains uncertain. Most studies believe the protective effect of ApoE2 on CHD [19,50,51,52], while others show ambiguous and opposite opinions [51,53,54,55]. The rarity of ApoE2 homozygous as ideal study objects makes it hard to conclude. Therefore, the application of transgenic animal models is necessary, and provides valuable data in the studies of disease relevancy, cellular and molecular mechanisms, and intervention strategies. Our study suggests that the ApoE2 isoform positively affects energetic production and cardiac contractility, but does not answer if ApoE2 shows a therapeutic effect on cardiac diseases, such as CHD and myocardial infarction. It will be significant to perform additional experiments to answer this question using disease models generated on hApoE KI rats. Recently, a clinical trial of adeno-associated virus (AAV) gene transfer vector-mediated ApoE2 expression as an AD therapeutic approach is ongoing at Cornell University (Phase 1: NCT03634007) to deliver the protective ApoE2 gene to the central nervous system for AD treatment. It will significantly benefit public health if similar research could be performed to investigate the potential treatment effect of ApoE2 on CHD and myocardial infarction. In addition, the ApoE2 allele frequency is significantly increased in centenarians group [16]. hApoE2 mice also show increased longevity associated with preserved activity (locomotor and exploratory activity) [17]. The effect of ApoE polymorphism on cardiovascular ageing warrants to be further investigated.

Animal models are undoubtedly valuable for biomedical research, yet whether animal models are predictive for humans is still disputable. Congruence was found to some extent on the effects of ApoE polymorphism on AD-related pathways between human studies, transgenic mouse models, and in vitro cell culture models [56]. However, no clear conclusion is made on the order of increased cardiac pathology for the ApoE isoforms. Mainstream studies indicate the cardiac risk association order of ApoE isoforms is E4 > E3 > E2 in men [12,19,21,50,51,52]. We found a positive effect of ApoE2 isoform on cardiac function and health in humanized rat models, which is consistent with population data. Interestingly, a significant association between LV dysfunction and the presence of the human ApoE3 was found in our rat model, different from the current knowledge that biochemical and biophysical characterization of endogenous rat ApoE is extended though like-human ApoE3 isoform [57]. Although mice carrying different human ApoE isoforms have been available for over two decades [58,59,60], study focusing on their cardiac function is still blank. Investigation regarding cardiac phenotypes using humanized mouse models with different ApoE isoforms and a study aiming to reveal the mechanisms of reduced heart function of hApoE3 rats are warranted. One bold guess about the incongruent results found in cardiac health between hApoE KI rats and men will be the amino acid disparateness on sites 112 and 158, two positions differing three human ApoE isoforms between humans and rodents such as mice and rats [61]. Mice and rats have arginine on both positions of their ApoE, the same as human ApoE4 instead of ApoE3. The minor difference determines the protein structure and function. Among the mammals, rabbit and bovine ApoE may be more appropriate to be considered as the “default” human ApoE3 isoform. Therefore, more studies need to be conducted using other humanized ApoE knock-in animal models.

Our study points out the two possible molecular mechanisms contributing to the enhanced heart contractile function of hApoE2 rats, but other mechanisms could be contributors as well. Another major limitation of this study is the limited availability of hApoE KI rats. Further studies need to be performed to validate ApoE2 protective function against cardiovascular ageing and the underlying mechanisms.

## 5. Conclusions

In summary, our study indicated that humanized rats expressing different human ApoE isoforms showed different cardiac functions. hApoE2 rats showed the optimized contractile function of the heart. In contrast, hApoE3 rats, similar to ApoE KO rats, exhibited impaired heart contractile function compared to WT and hApoE4 rats. Sufficient energy supply as the driving force, and adequate intracellular Ca^2+^ as the assistant secondary message, might explain the enhanced cardiac contractility of hApoE2 rats. Our studies suggested that human ApoE polymorphism is associated with heart performance, and could be a potential therapeutic target to prevent and treat heart diseases. Further studies are required to demonstrate the protective role of human ApoE2 in cardiac disorders.

## Data Availability

Data are available from the corresponding author on reasonable request.

## Supplementary Materials

The following supporting information can be downloaded at: https://www.mdpi.com/article/10.3390/cells12030347/s1, Table S1: Summary of antibodies; Supplementary Figure S1: Body weight and heart weight of transgenic rats at 6-month age. Supplementary Figure S2: Expression of mitochondrial proteins.
