# Supplementary material for: Human ApoE2 Endows Stronger Contractility in Rat Cardiomyocytes Enhancing Heart Function
Source: Cells. 2023 Jan 17;12(3):347. doi: 10.3390/cells12030347 (PMC9913850; doi:10.3390/cells12030347)
Supplement: Supplementary file 1 [file cells-12-00347-s001.zip › cells-2095002-supplementary.pdf]

Supplementary materials for the paper: Human ApoE2 endows stronger contractility in rat cardiomyocytes enhancing heart function

Yang Wu[1*], Fujie Zhao[1], Venkata N. Sure[1], Abdulgafar Ibrahim[1], Changjiang Yu[1], Sean M. Carr[1], and Ping Song[1*]

[1] Center for Molecular and Translational Medicine, Georgia State University, Atlanta, GA 30303, USA

[*] Correspondence: Yang Wu, dawn0722@gmail.com, 1-404-775-3434;
Ping Song, psong@gsu.edu, Tel.: +1-404-413-6636.

Table S1. Summary of antibodies.

Name of antibody	Company	Catalog #	Stain/blot	Source	Dilution for WB	Dilution for IF
CF555-WGA	Biotium	29076-1	Cell membrane			5µg/ml
DyLight[TM] 554 Phalloidin	Cell Signaling	13054	F-actin			1:200
ATP5A1	Proteintech	66037-1-lg	Mitochondria	mouse		1:150
AF 488, donkey anti-mouse	Invitrogen	A-21202	Secondary antibody	donkey		1:400
β-MHC	Santa Cruz Biotechnology, INC	sc-53090	223 kDa	mouse	1:500	
ANP	Santa Cruz Biotechnology, INC	sc-515701	17 kDa	mouse	1:500	
β-actin	Santa Cruz Biotechnology, INC	sc-47778	42 kDa	mouse	1:500	
MYH6	Abcam	ab207926	220 kDa	mouse	1:1000	
MYL2	Cell Signaling	3672	18 kDa	rabbit	1:1000	
TNNC1	Proteintech	13504-1-AP	18 kDa	rabbit	1:1000	
α-actin	Santa Cruz Biotechnology, INC	sc-32251	42 kDa	mouse	1:500	
GAPDH	Santa Cruz Biotechnology, INC	sc-32233	37 kDa	mouse	1:1000	
rApoE	Abcam	Ab183596	35 kDa	rabbit	1:1000	
hApoE	Cell Signaling	13366	35 kDa	rabbit	1:1000	
FAT/CD36	Cell Signaling	14347	88 kDa	rabbit	1:1000	
CPT1B	Millipore Sigma	SAB5700834	87 kDa	rabbit	1:1000	
COX IV	Proteintech	11242-1-AP	17 kDa	rabbit	1:5000	
Hsp60	Cell Signaling	2165	60 kDa	rabbit	1:1000	
VDAC1	Cell Signaling	4661	32 kDa	rabbit	1:1000	
Tim23	Santa Cruz Biotechnology, INC	sc-514463	23 kDa	mouse	1:1000	
Tom20	Santa Cruz Biotechnology, INC	sc-17764	17 kDa	mouse	1:500	
OPA1	Cell Signaling	80471	80-100 kDa	rabbit	1:1000	
Mfn1/2	Abcam	Ab57602	75 kDa	mouse	1:1000	
Mfn2	Cell Signaling	9482	80 kDa	rabbit	1:1000	
MFF	Santa Cruz Biotechnology, INC	sc-398617	25 kDa	mouse	1:500	
Fis1	Santa Cruz Biotechnology, INC	sc-376447	17 kDa	mouse	1:500	
Drp1	Cell Signaling	4494	84 kDa	rabbit	1:1000	
CACNA1C	Santa Cruz Biotechnology, INC	sc-398433	200 kDa	mouse	1:250	
RyR2	Santa Cruz Biotechnology, INC	sc-376507	565 kDa	mouse	1:250	
SERCA2	Santa Cruz Biotechnology, INC	sc-376235	109 kDa	mouse	1:250	

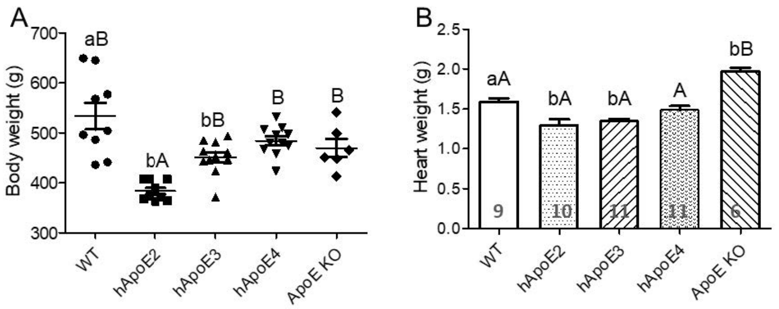

Supplementary Figure S1. Wu et al. Body weight and heart weight of transgenic rats at 6-month age. **A.** Body weight was compared between WT, hApoE2, hApoE3, hApoE4, and ApoE KO rats. **B.** Heart weight was compared between different rats. a vs. b, A vs. B, $p<0.05$. Rats number used for statistical analysis was labeled in bar graphs or indicates by symbols in scatter plots.

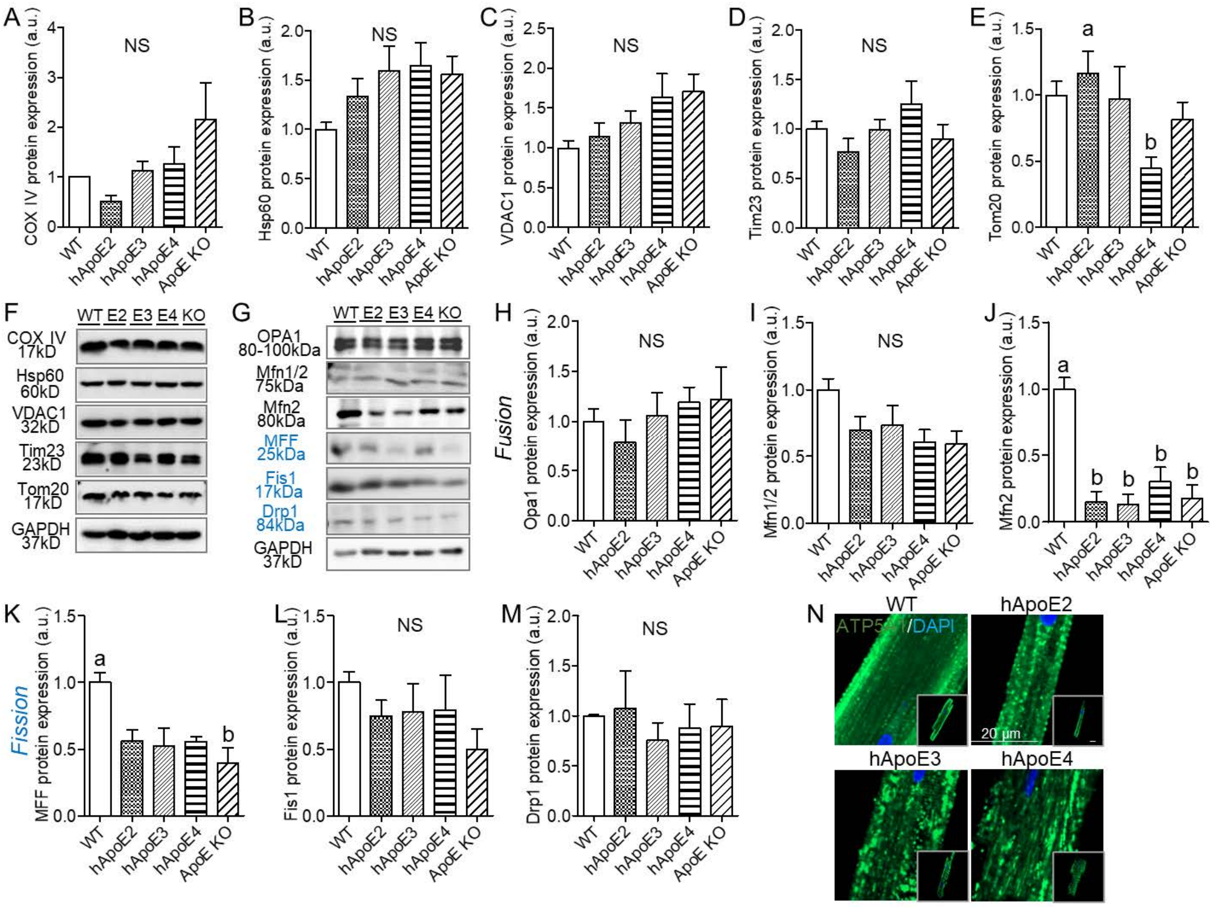

Supplementary Figure S2. Wu et al. Expression of mitochondrial proteins. **A-E.** A set of mitochondrial proteins, including COX IV, Hsp60, VDAC1, Tim23, and Tom20, were detected by western blotting using specific antibodies. **F.** Representative images of mitochondrial protein bands detected by western blotting were shown. **G.** Representative images of western blotting showed the expression patterns of proteins controlling mitochondrial dynamics. **H-M.** The expression profiles of proteins controlling mitochondrial fusion (Opa1, Mfn1, and Mfn2) and fission (MFF, Fis1, and Drp1) were quantified and compared in heart lysates from adult rats. **N.** Mitochondrial ATP synthase (Complex V) was labeled by anti-ATP5A1 antibody followed by a secondary antibody in Green, indicating the distribution of mitochondria in rat cardiomyocytes. Nuclei were stained by DAPI in blue. ANOVA followed by a Tukey post hoc test. a vs. b, $p<0.05$, NS, no significant difference. Six rats per group were used for western blotting analysis.
